# The relationship between language proficiency and attentional control in Cantonese-English bilingual children: evidence from Simon, Simon switching, and working memory tasks

**DOI:** 10.3389/fpsyg.2014.00954

**Published:** 2014-09-03

**Authors:** Chi-Shing Tse, Jeanette Altarriba

**Affiliations:** ^1^Department of Educational Psychology, The Chinese University of Hong KongHong Kong, China; ^2^Department of Psychology, University at Albany, State University of New YorkAlbany, NY, USA

**Keywords:** attentional control, bilingualism, conflict resolution, goal maintenance, working memory

## Abstract

By administering Simon, Simon switching, and operation-span working memory tasks to Cantonese-English bilingual children who varied in their first-language (L1, Cantonese) and second-language (L2, English) proficiencies, as quantified by standardized vocabulary test performance, the current study examined the effects of L1 and L2 proficiency on attentional control performance. Apart from mean performance, we conducted ex-Gaussian analyses to capture the modal and positive-tail components of participants' reaction time distributions in the Simon and Simon switching tasks. Bilinguals' L2 proficiency was associated with higher scores in the operation span task, and a shift of reaction time distributions in incongruent trials, relative to congruent trials (Simon effect in μ), and the tail size of reaction time distributions (τ) regardless of trial types in the Simon task. Bilinguals' L1 proficiency, which was strongly associated with participants' age, showed similar results, except that it was not associated with the Simon effect in μ. In contrast, neither bilinguals' L1 nor L2 proficiency modulated the global switch cost or local switch cost in the Simon switching task. After taking into account potential cognitive maturation by partialling out the participants' age, only (a) scores in the working memory task and (b) RT in incongruent trials and (c) Simon effect in μ in the Simon task could still be predicted by bilinguals' L2 proficiency. Overall, the current findings suggest that bilingual children's L2 proficiency was associated with their conflict resolution and working memory capacity, but not goal maintenance or task-set switching, when they performed the cognitive tasks that demanded attentional control. This was not entirely consistent with the findings of college-age bilinguals reported in previous studies.

## Introduction

The abilities to speak, write, read, and comprehend two languages are typically thought of as linguistic skills, but it has been reported that bilinguals outperform monolinguals in nonverbal tasks that demand attentional control (see, e.g., Bialystok et al., 2009; Hilchey and Klein, 2011, for reviews). Some researchers attributed this bilingual advantage in attentional control to their greater experiences in managing two language systems. When processing languages, bilinguals' first and second language (L1 and L2) systems create a conflict for selection, so they need to continuously monitor attentional resources to the target language (*goal maintenance*) and inhibit unwanted language to avoid confusion in language processing (*conflict resolution*) (e.g., Tse and Altarriba, 2012). Bilinguals also need to effectively switch between their two languages (*task-set switching*) during conversations in daily life. Experiences with ongoing demands of switching between two languages lead bilinguals to greater practice coordinating attentional resources and in turn enhance their task switching performance even though the tasks do not involve any verbal stimuli, although this claim has still been debated (see, e.g., Calabria et al., 2012). In the current study, we investigated the relationship between bilinguals' L1 and L2 proficiency and their performance in various cognitive tasks, including two nonverbal attentional control tasks (Simon and Simon switching) and one working memory task (operation span) within a homogeneous population (Cantonese, L1-English, L2 bilingual children). Before reviewing the evidence for or against the effect of bilingualism on attentional control, we introduce how performance on the current tasks could reflect one's conflict resolution, goal maintenance, and task-set switching abilities.

## Conflict resolution, goal maintenance, and task-set switching

In a typical trial of an arrow Simon task, an arrow appears either on the left or right side of the computer screen, and participants need to judge whether it is pointing to the left or right. Trials are said to be congruent when the arrow's direction and the arrow's location are consistent (left-pointing arrow appears on the left or right-pointing arrow appears on the right), whereas trials are said to be incongruent when the arrow's direction and the arrow's location are inconsistent (left-pointing arrow appears on the right or right-pointing arrow appears on the left). This task has often been used to test participants' attentional control abilities in previous studies (e.g., Bialystok et al., 2008; Tse et al., 2010). When participants respond to an incongruent trial (e.g., a right-pointing arrow appears on the left side of the screen), pathways that represent the arrow's direction and the arrow's location are engaged and compete for output. Participants must maintain the task goal (responding to the arrow's direction instead of the arrow's location) and resolve the conflict by suppressing the interference from the task-irrelevant arrow-location pathway and accessing the task-relevant arrow-direction pathway. This suppression does not occur in congruent trials where the arrow's direction and the arrow's location are congruent (right-pointing arrow appearing on the right side of the screen). The Simon effect occurs when participants respond slower and/or less accurately in the incongruent trials than in the congruent trials. Such effects reflect how well one can resolve the conflict between the arrow's direction and the arrow's location pathways. Those who show a smaller Simon effect possess stronger conflict resolution abilities. The participants' faster RT on the congruent trials could suggest that they are better at monitoring the task goal, even when the trial does not involve conflict resolution (see below for how components of the RT distribution reveal conflict resolution and goal maintenance abilities). The distinction between conflict resolution and goal maintenance is similar to the components in some other accounts, such as reactive (inhibition) control and proactive (monitoring) control in Morales et al. (2013b), which are briefly considered in the Discussion Section.

It is important to point out the distinction between the arrow Simon task that we used in the current study and the “color-square” Simon task used in other bilingual advantage studies (e.g., Bialystok et al., 2004). Following Kornblum's (1994) Dimensional Overlap Model (see also Blumenfeld and Marian, 2014), attentional control demanded in the arrow Simon task is similar to that involved in the Stroop task. Specifically, an arrow pointing to the right but appearing on the left side of the screen can create conflict between two overlapping perceptual dimensions (right direction vs. left location), similar to the conflict that occurs in the Stroop task (reading “red” printed in green color). Hence, the Simon effect in the current task could be attributed to the stimulus-stimulus conflict (i.e., conflict between two dimensions of the same stimulus), the stimulus-response conflict (i.e., conflict between stimulus dimension and response dimension), or both. This is different from the “color-square” Simon task that requires participants to respond to the color of a square (by pressing the key on the left or right side of the keyboard) that appears on the left or right side of the screen (see, e.g., Bialystok et al., 2004). Since there is no overlap between perceptual dimensions of the same stimulus (stimulus color and stimulus location), only stimulus-response conflict, but not stimulus-stimulus conflict, occurs in this type of Simon task. Previous studies showed that bilingual advantages more likely occurred in tasks that involved both stimulus-stimulus and stimulus-response conflicts (e.g., Bialystok et al., 2008; Carlson and Meltzoff, 2008), but are less likely to occur in those that involve only the stimulus-response conflict (e.g., Bialystok et al., 2005; Bialystok, 2006). Using these two types of tasks with other factors kept constant (e.g., stimuli), Blumenfeld and Marian revealed that bilinguals showed better performance in tasks that involved the stimulus-stimulus conflict than those that involved the stimulus-response conflict. Relatively speaking, monolinguals showed smaller differences in their performance between the two tasks. The current study was not designed to test between the bilingual advantages in the two types of Simon task. However, to maximize the potential effect of L2 proficiency and to compare our findings with those that used the Stroop task in the past (e.g., Tse and Altarriba, 2012), we used the arrow, rather than the color-square Simon task, in our current research.

The Simon switching task was used to examine participants' task-set switching abilities and, albeit indirectly, working memory capacities. The arrow stimuli were presented in either red or green, and participants responded to the arrow's location when it was in red or the arrow's direction when it was in green. All arrows were in red in the block of pure arrow-location trials and in green in the block of pure arrow-direction trials. These trials demonstrated performance when participants do not need to switch between the two task sets. In the mixed block, the arrow-direction and arrow-location trials were intermixed in a predictable alternating runs pattern (**A**A**B**B**A**A**B**B, see Rogers and Monsell, 1995). Participants responded with the same task set in mixed-nonswitch trials (the A and B in the above sequence) or switched between two task sets in mixed-switch trials (the **A** and **B** in the above sequence). To respond properly in the mixed block, they had to maintain both task sets in working memory, monitor the immediate demand of specific task requirements, and select the appropriate task set for their response (e.g., Hilchey and Klein, 2011). Global switch cost (RT/error difference between mixed-nonswitch trials and pure trials) reflects abilities to maintain both task sets in working memory and monitor the immediate demand of a constant switching requirement (e.g., Los, 1996). Local switch cost (RT/error difference between mixed-switch trials and mixed-nonswitch trials in the mixed block) reflects participants' abilities to suppress the irrelevant task set that was activated on previous trials, reactivate the relevant task set, and reconfigure via updating stimulus-response mapping according to the new task set over time (e.g., Monsell, 2003). (In the current study, the RT/error for global and local switch cost was all computed based on the pure, mixed-switch, and mixed-nonswitch trials, averaged across the arrow-direction trials and arrow-location trials.) Given the need to keep in mind the two task sets and monitor their demand on both switch and nonswitch trials in the mixed block, working memory load and monitoring demand may be the same for these two types of trials. Thus, local switch cost could be interpreted to reflect participants' task-set switching abilities, with their monitoring demand and working memory load being taken into account (see Koch et al., 2010 for a review). Following these interpretations, it could be argued that a smaller global switch cost may indicate better top-down management of competing task sets, thus reflecting stronger goal maintenance and higher working memory capacities, whereas a smaller local switch cost may reflect superior task-set switching abilities.

## Evidence for or against monolingual vs. bilingual differences in attentional control

Previous studies investigated the monolingual vs. bilingual performance differences in various cognitive tasks (e.g., Stroop and Attention Network Test) and reported the supporting evidence for the effect of bilingualism on conflict resolution (e.g., Bialystok et al., 2008; Carlson and Meltzoff, 2008; Costa et al., 2008; Bialystok and Feng, 2009; Bialystok, 2010; Marzecová et al., 2013; Pelham and Abrams, 2014), goal maintenance (e.g., Costa et al., 2008, 2009; Colzato et al., 2008; Martin-Rhee and Bialystok, 2008; Hilchey and Klein, 2011), working memory (despite being examined indirectly via a working memory load manipulation, e.g., Bialystok et al., 2008; Hernández et al., 2012; Morales et al., 2013a, or via a reduced global switch cost, e.g., Barac and Bialystok, 2012), and task-set switching (e.g., Costa et al., 2008; Garbin et al., 2010; Prior and MacWhinney, 2010). However, some studies reported a null effect of bilingualism on conflict resolution (e.g., Hilchey and Klein, 2011; Kousaie and Phillips, 2012; Paap and Greenberg, 2013), goal maintenance (e.g., Kousaie and Phillips, 2012; Paap and Greenberg, 2013), working memory (e.g., Bonifacci et al., 2011) or task-set switching (e.g., Hernández et al., 2013). Moreover, some researchers argued that the effect of bilingualism could be modulated by various factors, such as socioeconomic status (e.g., Morton and Harper, 2007; Gathercole et al., 2010; but see Yang et al., 2011; Engel de Abreu et al., 2012; Calvo and Bialystok, 2014).

## Evidence for the role of language proficiency in conflict resolution and goal maintenance within bilinguals

To minimize the potential problems associated with the differences between monolinguals and bilinguals (e.g., socioeconomic status), some studies examined attentional control abilities within the bilingual population. Carlson and Meltzoff (2008) showed that after controlling for age, parents' education, and group differences in overall RTs, balanced bilingual children performed better than unbalanced bilingual children in tasks that demand conflict resolution. Luo et al. (2010) found that young-adult bilinguals with higher vocabulary knowledge demonstrated better performance in letter fluency, which may tap conflict resolution and goal maintenance, than those with lower vocabulary knowledge. Using the Attention Network Task, Tao et al. (2011) found that college-age bilinguals with more balanced L1 and L2 proficiency and usage showed better conflict resolution abilities. Luk et al. (2011) reported that a later onset age of active bilingualism in young adults was associated with a smaller L2 vocabulary size and lower conflict resolution abilities, as indicated by a stronger interference effect in a flanker task. Similarly, by testing bilingual children in an L2 immersion program, Bialystok and Barac (2012) observed that the interference effect in a flanker task was weaker when they received more years of bilingual education and/or had more balanced L1/L2 proficiencies. Videsott et al. (2012) reported that multilingual children with higher linguistic competence were better at detecting the target stimulus than those with lower linguistic competence in the Attention Network Task. Heidlmayr et al. (2014) tested bilinguals who ranged in age from 25 to 35 years old and showed that those bilinguals with longer duration of L2 immersion performed better on the Stroop task. All these studies converged upon the conclusion that at least some components of attentional control may be enhanced by bilinguals' L1/L2 proficiencies, regardless of their ages and types of L1 and L2.

A few recent studies have employed RT distributional analyses to identify the underlying attentional control mechanisms that are positively associated with bilinguals' L2 proficiency (see Tse and Altarriba, 2012 for a more detailed introduction to these analyses and a brief review of studies that used this analytic technique to examine attentional control). Analyses of RT distributional characteristics can be done by fitting individual raw RT to a theoretical ex-Gaussian distribution that closely approximates the typical positively-skewed empirical distribution. This distribution is defined by a three-parameter function: μ and σ reflect the mean and standard deviation of the Gaussian component, respectively, and τ reflects the exponential component of the RT distribution. The ex-Gaussian analyses can be used as a descriptive model for capturing the influence of a variable on RT distributions, with the parameters having a direct relation to the mean of a distribution. The algebraic sum of μ and τ is constrained to approximate closely the empirical distribution, so the difference in mean RT between two conditions can be partitioned to be distributional shifting and/or a change in the tail of the RT distribution. A longer RT due to a group difference or a manipulation could be attributed to a shift of the RT distribution, an increase in the tail size of the RT distribution, or both.

In the selective attention task the μ and τ estimated on the basis of participants' RT distribution have implications for the efficiency of their attentional control abilities. Take the arrow Simon task as an example. A shift due to the arrow's direction vs. arrow's location congruency in the RT distribution (μ difference in incongruent vs. congruent trials—Simon effect in μ) can be due to the fact that incongruent trials demand conflict resolution in the form of the inhibition of a response to the arrow's location and the generation of a correct response to the arrow's direction. This adds about a constant amount of time to incongruent trials, making them on average slower than congruent trials. The smaller the Simon effect in μ, the better one resolves the conflict between information from different sources. In contrast, transient failures of goal maintenance lead to very slow correct RT as indexed by a lengthening tail of the RT distribution (larger τ) based on all trials of the task. The better the participants' goal maintenance abilities, the less likely they lose their task goal but then correct before an overt error is produced, the smaller proportion of very slow correct RT they show, and in turn, the shorter the tail of their RT distribution (i.e., the smaller the τ).

Using a Stroop color-naming task, Tse and Altarriba (2012) reported that both conflict resolution (as reflected by a shift of RT distribution in incongruent trials, relative to congruent trials, i.e., Stroop effect in μ) and goal maintenance (as reflected by a lengthening of the tail of an RT distribution, i.e., a change in τ) were better when bilingual's L1 and/or L2 proficiencies were higher. However, Calabria et al. (2011) reported inconsistent results. Using a nonverbal flanker task with key-press responses, they found that the bilingual vs. monolingual difference in the congruency effect lay in the tail size of the RT distribution (congruency effect in τ), but not in the shift of the RT distribution (congruency effect in μ), as reported in Tse and Altarriba.

Given that there were a number of procedural differences in Tse and Altarriba (2012) and Calabria et al. (2011), it is not clear whether the findings of these two studies could be directly compared. Nonetheless, we highlighted two of them to motivate the current research. First, it is possible that the Stroop task in Tse and Altarriba involved vocal responses and thus was more likely to tap participants' language abilities (than Calabria et al.'s nonverbal flanker task). While these authors did control this by treating participants' overall RT in the baseline blocks (in which they only read aloud the color names or the ink color of color patches) as covariates in their analyses, it is not clear whether the influence of language on performance in the baseline block, where no conflict resolution or high demand of goal maintenance was required, could directly reflect the influence of language on performance in the actual Stroop color-naming task. Hence, it is important to use a task with a minimal language requirement, as was Calabria et al.'s flanker task. To achieve this, we used a non-verbal Simon task with arrow stimuli in the current study. In addition, similar to Tse and Altarriba's Stroop task and Calabria et al.'s flanker task, our arrow Simon task is sensitive to both stimulus-stimulus conflict and stimulus-response conflict (see Blumenfeld and Marian, 2014, for more details), so the findings of the current study could be viewed as comparable to those reported in these two studies.

Second, whereas Tse and Altarriba (2012) used a heterogeneous pool of bilinguals who had a wide range of L1 but the same L2 (English), Calabria et al. (2011) used a homogeneous pool of Catalan-Spanish bilinguals and Spanish monolinguals, with the two languages (Catalan and Spanish) being highly similar in their orthographies. Prior studies showed that cross-linguistic script similarity could modulate the bilingual advantage in attentional control (e.g., Coderre et al., 2013; Hernández et al., 2013), so it is not clear to what extent this factor contributed to the discrepancy of the findings between the two studies. Also, Tse and Altarriba did not assess their participants' nonverbal intelligence, so their findings could be clouded by the possibility that bilinguals with higher L1/L2 proficiencies tended to have higher nonverbal intelligence, which in turn contributed to their superior attentional control abilities. To address these concerns, the current study used a homogeneous pool of Cantonese-English bilingual children, who have the same L1 and L2, and directly measured their L1/L2 proficiency and nonverbal intelligence using standardized tests to further investigate whether Tse and Altarriba's results, which were based on a college-age population, would generalize, to a child population.

## Evidence for the role of language proficiency in task-set switching and potential effects of language proficiency on working memory within bilinguals

Some studies have examined the effect of bilingualism on task-set switching within bilingual populations. While these studies targeted task switching performance, as mentioned above, their findings of a global switch cost could also shed light on the effect of bilingualism on working memory. Prior and Gollan (2011) found that young-adult bilinguals who claimed to switch between two languages more often showed smaller local switch costs yet equivalent global switch costs in both language and task-set switching than those who claimed to switch less frequently (see similar findings reported in Tse and Altarriba's in press, Stroop switching task when bilingualism was defined by self-rated L2 proficiency). This could suggest a positive effect of bilingualism on task-set switching, but not on working memory. On the other hand, Soveri et al. (2011) found in a number/letter switching task that there was a negative correlation between language-switching frequency and global switch cost in errors (but not in local switch cost) in bilinguals ranging in age from 30 to 75 years old. This could suggest a positive effect of bilingualism on working memory, but not on task-set switching. Some studies manipulated working memory load and reported a lesser influence of these loads in bilinguals (relative to monolinguals) on their task performance (e.g., Bialystok et al., 2008; Hernández et al., 2012; Morales et al., 2013a). Nonetheless, the effect of bilingualism on working memory in these studies has merely been inferred, without actually measuring their working memory capacity. Moreover, previous evidence for the monolingual vs. bilingual difference in working memory capacity has also been mixed. By applying confirmatory factor analyses to the data of 12 working memory tasks, Soliman (2014) found that the componential structures of a working memory model (Baddeley and Logie, 1999) were similar in monolinguals and bilinguals, and that bilinguals yielded higher latent factor means than (i.e., outperformed) monolinguals in all four components as defined in Baddeley and Logie's model: phonological loop, central executive, visuospatial sketch, and episodic buffer. Kaushanskaya et al. (2014) also found that bilingual children, who acquired the L2 in classroom settings for about 2 years, performed better than monolingual children in a verbal working memory task, although the two groups did not differ in short-term memory performance or nonverbal task-set switching. Nguyen and Astington (2014) showed that after controlling for language proficiency and age, bilingual children outperformed monolingual children in a backward span task. Contrary to these studies that provided positive evidence, Luo et al. (2012; see also Morales et al., 2013a) reported that relative to young-adult monolinguals, young-adult bilinguals showed better performance in spatial working memory, but worse performance in verbal working memory. Bonifacci et al. (2011) even failed to find any bilingual vs. monolingual performance differences in children on the non-verbal (number and symbol) working memory tasks. To our knowledge, no published study has examined the relationship between bilinguals' L1/L2 proficiency and working memory capacity using a standard complex memory span task, such as operation span (e.g., Unsworth et al., 2005). Rather than finding out the components of working memory that could be most affected by bilinguals' L1/L2 proficiency (e.g., Soliman, 2014), we tapped bilinguals' general working memory capacity by using the operation span task in the current research and examined whether it could be correlated with L1/L2 proficiency. This task, rather than reading span task, was chosen because previous studies showed that the high language requirement in the reading span task could make it less likely to reflect bilinguals' genuine working memory capacity than the operation span task (e.g., Sanchez et al., 2010).

## Present research

To recapitulate, in the current study we had a group of Cantonese-English bilingual children perform the arrow Simon task, Simon switching task, and operation span task to clarify the inter-relationship among global and local switch costs, task-set switching, and working memory capacities, as well as their associations with L1/L2 proficiency within the bilingual population. If conflict resolution, goal maintenance, task-set switching, and working memory could all be modulated by bilinguals' L1/L2 proficiency, we predicted that after controlling for demographic variables like participants' age (which could reflect the age-related cognitive maturation), socioeconomic status, and nonverbal intelligence, relative to those with lower L1/L2 proficiencies, bilinguals with higher L1/L2 proficiencies would show:

1. A faster overall RT in congruent and incongruent trials, a smaller Simon effect, a smaller RT distribution tail (as reflected by τ) whether it was based on overall RT or on individual trial types (e.g., congruent trials), and a smaller shift of RT distribution in the incongruent vs. congruent trials (as reflected by Simon effect in μ) in the Simon task.
2. A smaller local switch cost and a smaller global switch cost in the Simon switching task.
3. A higher score in the operation span task.

## Methods

### Participants

We recruited 100 Cantonese-English bilingual children (ages 5–9) from local kindergartens and elementary schools in Hong Kong and obtained informed consents from their parents, prior to their participation. The current study was approved by the Chinese University of Hong Kong Survey and Behavioral Research Ethics Committee. Because the number of participants was not evenly distributed in the five age groups, we did not examine age-related differences in the relationship between attentional control and language proficiency in the current research. For the sociolinguistic environment of the participants, the media of instruction were Cantonese and English of about equal proportion in their school setting. Communication between the children and their parents was mainly through Cantonese at their home. Table 1 presents the demographic information for participants. On average, participants began to learn English letters and began reading English in the pre-nursery schools or kindergartens at about 2–3 years of age. The standard deviations of the Chinese and English vocabulary scores indicated that participants' L1/L2 proficiencies were quite diverse. Their nonverbal intelligences were high on average. Socioeconomic status was computed based on the parents' highest level of education and their total annual income. Each parent received a score (1–4) based on their level of academic achievement (e.g., junior high or less=1; postgraduate=4). Families received a score (1–10) based on their annual household income (e.g., less than HKD 160 k = 1; HKD 160 k- HKD 320 k = 2; etc.). Two parent education scores and income scores were averaged to create the socioeconomic status score.

**Table 1:** **Mean, standard deviation (*SD*), and range for participants' demographic information**.

	**Mean**	***SD***	**Range**
Age	6.09	1.45	5–9
Proportion of sex (M:F)	50:50		
Proportion of handedness (left:right)	12:88		
Age of acquisition for English	2.52	1.31	0–8
Socioeconomic status	3.32	1.14	1–6
Chinese vocabulary scores	24.33	12.61	4–65
English vocabulary scores	4.93	2.82	1–15
Nonverbal intelligence standardized scores	121.85	13.55	85–150
Nonverbal intelligence percentiles	85.99	17.81	16–100

*See Methods Section for the details of the scales and definitions of socioeconomic status. All participants' parents mentioned that their children began to learn Cantonese since birth*.

### Materials and procedures

PC-compatible laptop computers with E-Prime were used to display stimuli and collect RT and error data. Participants were tested in groups of 10–30 for the Raven's Standard Progressive Matrices task and individually in a classroom for the other tasks. To ensure the reliability of the group administration of the Raven's Standard Progressive Matrices task (e.g., participants took the task seriously and did not talk to each other during the task), the research assistant and two well-trained student helpers (who were college students at the Chinese University of Hong Kong) closely monitored the process of data collection to ensure that all participants followed the task instructions. There were two days of testing sessions. All participants performed the Raven's Colored Progressive Matrices, Simon task, and Chinese Vocabulary Definition Test on the first day, and Simon switching task, working memory task, and Raven's Crichton Vocabulary Scale on the second day. The participants received verbal instructions in Cantonese in all tasks.

#### Raven's colored progressive matrices

In this untimed task, there were 36 items arranged in 3 sets of 12 items each. Each item contained a logical pattern with a missing piece. Below the pattern, there were 6 possible pieces to complete the pattern. The sets and the items within the sets were presented in order of difficulty. The participants were given a score for each correct answer, and these raw scores were converted into standardized scores based on their ages. We chose this task as it is highly reliable (0.70–0.90), correlated with other intelligence tests (0.50–0.75) (Murphy and Davidshofer, 2005), and does not tap language skills (Kaplan and Saccuzzo, 2009). A similar version was used in previous bilingual studies (e.g., Colzato et al., 2008).

#### Chinese vocabulary definition test

The test is comprised of 53 Chinese vocabulary items. We used the test procedures and scoring scheme of the Hong Kong Wechsler Intelligence Scale for Children (Hong Kong Education Department and Hong Kong Psychological Society, 1981) to score participants' definitions. Sample answers for zero to two points per question were also included in the scoring key as well as examples given for each item [e.g., policemen, 0 = nothing relevant (“those with uniform”); 1 = somewhat relevant yet incomplete (“person who catches people”); 2 = relevant (“person who catches thieves/bad guys, with guns and handcuffs”)]. The maximum score was 106. The researcher presented each item orally and the participant explained objects/concepts of increasing conceptual difficulty. The researcher was trained on this key in rating participants' answers, and the test stopped when s/he obtained a score of zero across 5 consecutive items. The test was used in previous studies conducted in Hong Kong (e.g., McBride-Chang et al., 2008).

#### Raven's Crichton vocabulary scale

This procedure was similar to the Chinese Vocabulary Definition Test, except that this test comprised 40 English vocabulary items. Because we adapted the same scoring procedure as in the Chinese test, the maximum score was 80. This test was used because its task demand matched that of the Chinese test; that is, each task required participants to explain the meaning of a set of vocabulary words.

#### Simon task

In this task, the stimulus display consisted of a white fixation point +++ on the screen and white arrow stimuli (measuring approximately 4 cm in length and 2 cm in height) presented on a black background. The peripheral locations of the arrow (left and right) were typically situated 5° on the horizontal plane from the central fixation. The participants were told that they would be presented with an arrow pointing to either the left or right on the screen. The arrow appeared on the left or right half of the screen. They were told to ignore the arrow's location on the screen and respond according to the arrow's direction by pressing either the left key or the right key of the response box. In the congruent condition, the arrow's direction corresponded to the arrow's location (e.g., left-pointing arrow on the left side of the screen). In the incongruent condition, the arrow's direction was opposite to the arrow's location (e.g., left-pointing arrow on the right side of the screen). Each trial began with a 500-millisecond (ms) central fixation point, followed by the onset of an arrow that stayed on the screen until the participant made a response or until 5 s had elapsed. Once a response was made, the screen cleared and a 400-ms message denoting the accuracy of their performance was presented. The program recorded participants' RT and accuracy. Following a 750-ms blank-screen intertrial interval, another trial began. To increase the sensitivity of the task in detecting the role of monitoring (cf. Costa et al., 2009), congruent and incongruent trials were in equal proportion and randomly intermixed, so there were 60 congruent and 60 incongruent trials, with the left- and right-pointing arrows appearing on the left or right side of the screen equally often. Prior to actual trials, participants were given 8 practice trials. There was a self-paced break midway through the task (i.e., after 60 trials). This task was shown to measure the same attentional control construct as the Stroop task did in previous research (e.g., Tse et al., 2010, see also Blumenfeld and Marian, 2014, for the rationale that the Stroop and arrow Simon tasks may measure similar types of conflicts). Given that no neutral trials (e.g., the arrow appearing at the center of the screen) was included in this task, we could not tease apart facilitation due to the congruency between the location and direction information and the interference due to the incongruency between the location and direction information that may have contributed to the Simon effect in the present study (see Tse and Altarriba, 2012, for a similar manipulation in the Stroop task).

#### Simon switching task

The stimuli and presentation procedures were identical to those in the Simon task, except that there were two types of trials. When the arrow was presented in green (direction trials), participants judged the arrow's direction (by pressing either the left or the right key on the response box). When the arrow was presented in red (location trials), they judged the arrow's location (by pressing either the left or the right key on the response box). There were three blocks of trials. In the first and second blocks (i.e., pure block), participants received only 48 direction and 48 location trials, respectively, whereas in the third block (i.e., mixed block), participants received 24 direction and 24 location trials, which were presented in an AABB alternating runs pattern. That is, the mixed block contained a total of 24 switch trials (12 for direction and 12 for location) and 24 non-switch trials (12 for direction and 12 for location). This paradigm allows for a comparison of performance on switch trials (AB, BA) with performance on non-switch trials (AA, BB) (cf. Rogers and Monsell, 1995). We compared the switch and non-switch trials within the same block to yield the local switch cost and compared the non-switch trials in the mixed block and all trials in the pure block to yield the global switch cost. Prior to actual trials in each block, participants were given 8 practice trials. There was a self-paced break midway through each block (i.e., after 24 trials).

#### Working memory task

We adapted Unsworth et al.'s (2005) operation span task, which shows good internal consistency (0.78) and test-retest reliability (0.83). On each trial, participants were presented with a series of letters (from the English alphabet), each of which was followed by a 2-operator arithmetic problem. As shown in our pilot data, the original version of this task was too difficult for our child participants, so we modified the stimuli such that the arithmetic problem only involved the addition or subtraction of 1 through 5. Across trials, the number of letters for memorization varied randomly from 2 to 7. At the end of a letter-arithmetic-problem sequence, participants recalled letters in the same order as appeared before. High scores can be achieved by holding more letters in memory, while maintaining pre-specified accuracy (85%) on the arithmetic task. We used the absolute operation-span scoring method to yield a working memory score (hereafter WM score) (see Unsworth et al. for the details of its task structure, scoring, and validity).

At the end of all tasks on Day 2, we required participants' parents to fill out a survey, in which we asked for their child's sex, age, handedness, age of acquisition for Cantonese and English, and socioeconomic status. All participants were debriefed at the end of the entire study.

## Results

The level of significance was set at 0.05, one-tailed, because all of our predicted effects were in specific directions. The effect sizes of *F* and *t* statistics are in η^2^_*p*_ and Cohen's *d* (hereafter *d*), respectively. Prior to the analyses, we performed the following preliminary data treatment for the Simon and Simon switching task. We first excluded RT for incorrect responses. For trials with correct responses, we excluded those that were shorter than 200 ms and >3 *SD* above and below each participant's overall mean (~3.0% for Simon task and ~3.3% for Simon switching task). The correlation between overall RT and errors was non-significant in the Simon and Simon switching tasks (+0.03 and +0.09, respectively, both *ps* > 0.05), indicating that there was no tradeoff between speed and accuracy. We examined individual raw RT distributions by estimating participants' ex-Gaussian parameters using a quantile maximum likelihood estimation procedure in QMPE 2.18, following previous studies (e.g., Tse and Altarriba, 2012). All fits successfully converged within 250 iterations. For the Simon task, we estimated ex-Gaussian parameters for overall RT and RT for congruent and incongruent trials. However, in the Simon switching task because there was a smaller number of observations for the switch trials and the nonswitch trials within the mixed block after the trials with incorrect responses or outlier RT were eliminated (i.e., fewer than the initial 24 trials per participant), we only estimated ex-Gaussian parameters for overall RT and RT of the pure and mixed blocks. Hence, we could not investigate the global and local switch cost in terms of ex-Gaussian parameters, which was not related to any of our predicted effects. Tables 2 and 3 present cell means and the significance of L1 and L2 proficiencies on predicting participants' performance in the Simon and Simon switching tasks.

**Table 2:** **Mean statistics and findings of regression analyses for participants' performance in the Simon task**.

		**Mean**	***SD***	***R^2^* change**	**L1 Proficiency**			**L2 Proficiency**		
					***beta***	***t*_(93)_**	***p***	***beta***	***t*_(93)_**	***p***
Overall	RT	874	278	0.18^*^	−0.28^*^	2.62	0.01	−0.23^*^	2.22	0.02
	Error	23	13	0.01	−0.11	0.93	0.18	0.02	0.19	0.43
	μ	632	170	0.10^*^	−0.20^*^	1.80	0.04	−0.18^*^	1.70	0.045
	σ	136	69	0.08^*^	−0.15	1.25	0.11	−0.17	1.51	0.07
	τ	244	166	0.16^*^	−0.27^*^	2.38	0.01	−0.20^*^	1.83	0.04
Simon effect	RT	112	114	0.04	0.06	0.48	0.32	−0.23^*^	1.94	0.03
	Error	16	17	0.00	0.01	0.09	0.47	−0.01	0.10	0.47
	μ	129	134	0.06^*^	0.15	1.24	0.11	−0.29^*^	2.45	0.01
	σ	9	96	0.03	0.19	1.50	0.07	−0.15	1.25	0.11
	τ	−16	124	0.01	−0.12	0.95	0.18	0.11	0.91	0.19
Congruent trials	RT	827	269	0.17^*^	−0.29^*^	2.67	0.01	−0.21^*^	2.00	0.02
	Error	15	11	0.01	−0.14	1.15	0.13	0.03	0.29	0.39
	μ	582	172	0.08^*^	−0.21^*^	1.90	0.03	−0.13	1.27	0.11
	σ	119	78	0.06^*^	−0.17	1.34	0.09	−0.12	1.03	0.15
	τ	247	155	0.17^*^	−0.27^*^	2.38	0.01	−0.21^*^	1.94	0.03
Incongruent trials	RT	938	300	0.18^*^	−0.24^*^	2.17	0.02	−0.27^*^	2.64	0.01
	Error	31	19	0.004	−0.07	0.58	0.28	0.01	0.08	0.47
	μ	711	192	0.13^*^	−0.08	0.78	0.22	−0.32^*^	3.14	<0.01
	σ	128	89	0.06^*^	0.06	0.46	0.33	−0.27^*^	2.30	0.01
	τ	231	184	0.13^*^	−0.31^*^	2.62	0.01	−0.10	0.93	0.18

* *p < 0.05 (one-tailed). The R^2^ change column shows the increase in R^2^ after adding L1 proficiency and L2 proficiency predictor variables and its significance. The L1 proficiency and L2 proficiency columns show the significance of the L1 proficiency and L2 proficiency predictor variables in the regression analyses, respectively*.

**Table 3:** **Mean statistics and findings of regression analyses for participants' performance in the Simon switching task**.

		**Mean**	***SD***	**R^2^ change**	**L1 Proficiency**			**L2 Proficiency**		
					***beta***	***t*_(93)_**	***p***	***beta***	***t*_(93)_**	***p***
Overall	RT	864	335	0.12^*^	−0.28^*^	2.44	0.01	−0.12	1.08	0.15
	Error	27	15	0.01	−0.09	0.73	0.23	−0.04	0.35	0.37
	μ	565	198	0.06^*^	−0.15	1.27	0.11	−0.15	1.33	0.10
	σ	118	86	0.05^*^	−0.13	1.04	0.15	−0.14	1.17	0.12
	τ	301	214	0.10^*^	−0.30^*^	2.55	0.01	−0.04	0.37	0.36
All trials in the pure block	RT	814	303	0.12^*^	−0.28^*^	2.42	0.01	−0.14	1.26	0.11
	Error	21	16	0.05^*^	−0.19	1.53	0.07	−0.06	0.56	0.29
	μ	560	184	0.06^*^	−0.17	1.45	0.08	−0.13	1.19	0.12
	σ	113	74	0.05^*^	−0.18	1.45	0.08	−0.10	0.83	0.21
	τ	257	188	0.11^*^	−0.29^*^	2.40	0.01	−0.10	0.84	0.20
All trials in the mixed block	RT	981	451	0.08^*^	−0.26^*^	2.20	0.02	−0.06	0.56	0.29
	Error	29	16	0.001	−0.02	0.14	0.45	−0.02	0.16	0.44
	μ	672	324	0.03	−0.15	1.20	0.12	−0.05	0.43	0.34
	σ	136	128	0.01	−0.07	0.54	0.30	−0.04	0.33	0.37
	τ	315	271	0.08^*^	−0.27^*^	2.29	0.01	−0.04	0.36	0.36
Switch trials in the mixed block	RT	1012	483	0.08^*^	−0.27^*^	2.26	0.02	−0.04	0.36	0.36
	Error	33	17	0.001	0.02	0.16	0.44	0.02	0.14	0.45
Nonswitch trials in the mixed block	RT	951	438	0.08^*^	−0.24^*^	2.05	0.02	−0.07	0.64	0.26
	Error	26	17	0.01	−0.05	0.39	0.35	−0.05	0.41	0.35
Global switch cost	RT	137	242	0.01	−0.10	0.75	0.23	0.04	0.34	0.37
	Error	5	13	0.03	0.16	1.33	0.10	0.02	0.15	0.44
Local switch cost	RT	62	200	0.01	−0.12	0.96	0.17	0.06	0.49	0.31
	Error	7	12	0.02	0.10	0.77	0.22	0.09	0.77	0.23

* *p < 0.05 (one-tailed). The R^2^ change column shows the increase in R^2^ after adding L1 proficiency and L2 proficiency predictor variables and its significance. The L1 proficiency and L2 proficiency column shows the significance of the L1 proficiency and L2 proficiency predictor variables in the regression analyses, respectively*.

### Overall performance in the Simon task, Simon switching task, and operation span task

In the Simon task, the Simon effects (RT/error differences in incongruent vs. congruent trials) were significant (both *ts* > 9.37, *p*s < 0.001, *d*s > 1.33). Analyses of ex-Gaussian parameters showed that the Simon effect in RT was due to a congruent vs. incongruent difference in μ [582 vs. 711, *t*_(99)_ = 9.63, *p* < 0.001, *d* = 1.36], but not in τ [247 vs. 231, *t*_(99)_ = 1.33, *p* = 0.09, *d* = 0.19]. This was analogous to the findings in previous studies (e.g., global-local congruency effect in Spieler et al., 2000; Stroop effect in Tse and Altarriba, 2012) that the Simon effect was attributed to an increase in μ, rather than τ, in incongruent trials, relative to congruent trials. Hence, the Simon effect in μ provided an index of conflict resolution for participants' performance in the Simon task.

In the Simon switching task, we obtained an overall Simon effect [*F*_(1, 99)_ = 37.02, *MSE* = 33659, *p* < 0.01, η^2^_*p*_ = 0.27]. However, this effect did not interact with global and local switch cost (all *F*s < 1.97, *p*s > 0.05). To avoid complexity in our analyses for issues that were not relevant to our hypothesis testing, we collapsed across the congruency variable and focused on switching performance in the following analyses. The overall global and local switch costs were significant in RT and errors (all *t*s > 3.10, *p*s < 0.01, *d*s > 0.44). Hence, even though the novel Simon switching task might be different in stimuli from those used in previous studies (e.g., Stroop switching), it demonstrated the typical findings in task switching literature.

In the working memory (operation span) task, participants' mean WM score was 22.48 (*SD* = 16.24). The standard deviation indicated that their working memory capacity was quite diverse.

### Correlation analyses among attentional control measures and demographic variables

First, the WM score negatively correlated with the overall τ more strongly than with the overall μ and overall σ in the Simon task (−0.43 vs. −0.33 and −0.26, all *p*s < 0.01; after partialling out participants' age, −0.23 vs. −0.07 and −0.12, *p* = 0.01, 0.25, and 0.12, respectively) and, albeit not as large in their differences, in the Simon switching task (−0.37 vs. − 0.33 and −0.31, all *p*s < 0.01; after partialling out participants' age, −0.20 vs. −0.12 and −0.18, *p* = 0.02, 0.12, and 0.04, respectively). This was consistent with the findings of previous non-bilingual studies in which τ was associated with working memory capacity more than μ and σ (e.g., Tse et al., 2010).

Second, it was possible that global switch cost may be associated with goal maintenance and working memory capacity because it may reflect the difference in participants' nonswitch trial performance when they need to maintain the two task sets in working memory and monitor their demand in the mixed block vs. when they do not need to do that in the pure block. We focused on the τ from congruent trials and pure-block trials because they could more clearly reflect goal maintenance abilities under the circumstance where no conflict resolution is needed. The current findings were partially consistent with these relationships. The global switch cost in RT was correlated with the τ in congruent trials in the Simon task (+0.34, *p* < 0.001; after partialling out participants' age, +0.33, *p* < 0.001) and with the τ in pure-block trials in the Simon switching task (+0.39, *p* < 0.001; after partialling out participants' age, +0.38, *p* < 0.001), but not with the Simon effect in μ in the Simon task (−0.06, *p* = 0.28; after partialling out participants' age, −0.07, *p* = 0.24). This suggests that the global switch cost was associated with one's goal maintenance, rather than conflict resolution abilities. In contrast, global switch cost was not correlated with WM scores (−0.04, *p* = 0.36; after partialling out participants' age, +0.03, *p* = 0.39), suggesting that it was not associated with one's working memory capacity.

Third, in the Simon task, bilinguals with higher L1 or L2 proficiencies responded faster in congruent and incongruent trials and yielded a smaller τ, whether the parameter was estimated based on overall RT or on the individual trial types (congruent or incongruent) (all *r*s < −0.24). However, after partialling out participants' age, only two correlation coefficients remained at least marginally significant: the relationship between L2 proficiency and τ in the congruent trials, *r* = −0.17, *p* = 0.04, and the relationship between L2 proficiency and τ in overall RT, *r* = −0.15, *p* = 0.07. On the other hand, only bilinguals' L2, but not L1, proficiency was negatively correlated with the Simon effect in RT (−0.20 and −0.07, *p* = 0.02 and 0.26, respectively, after partialling out participants' age, −0.15 and +0.11, *p* = 0.07 and 0.13, respectively) and the Simon effect in μ (−0.23 and −0.03, *p* = 0.01 and 0.37, respectively, after partialling out participants' age, −0.21 and −0.07, *p* = 0.02 and 0.24, respectively). In the Simon switching task, there was no relationship between bilinguals' L1 or L2 proficiencies and global or local switch cost, whether participants' age was or was not controlled in the analyses (all *r*s < 0.07, *p*s > 0.25). In the operation span task, bilinguals' with higher L1 or L2 proficiencies yielded higher WM scores (+0.51 and +0.50, respectively, both *p*s < 0.01, after partialling out participants' age, +0.37 and +0.18, *p* < 0.01 and *p* = 0.04, respectively).

### Regression analyses for the relationship between L1/L2 proficiency and attentional control

We performed multiple regression analyses to further examine whether L1/L2 proficiency could predict participants' performance in attentional control measures, after taking into account their demographic variables such as socioeconomic status. We entered participants' handedness, sex, nonverbal intelligence percentiles, and socioeconomic status in the first step and the Chinese and English vocabulary scores, which reflect bilinguals' L1 and L2 proficiencies, respectively, in the second step. These analyses allowed for an examination of whether bilinguals' L1 and L2 proficiencies could uniquely predict the variance of each dependent measure. To examine the relationship between L2 proficiency and attentional control after taking into account bilinguals' cognitive maturation, we repeated the above analyses by entering participants' age in the first step of the models. However, given the strong correlation between participants' age and L1 proficiency (*r* = +0.80), in these analyses only L2 proficiency was entered in the second step of the models. To control for the influence of L1 proficiency, we computed the ratio of L2 proficiency to L1 proficiency (L2:L1 ratio) for each participant and then repeated the above analyses by entering this ratio measure, rather than L2 proficiency, in the second step of the models^1^. By examining whether some of the significant L2 proficiency effects obtained in our first set of analyses could be eliminated by partialling out participants' age in the regression models, we could, albeit indirectly, test the contribution of cognitive maturation in the relationship between language proficiency and attentional control. Tables 2 and 3 show the results of the full models of the multiple regression analyses, whereas Tables 4 and 5 show the results of the full models in which participants' age was partialled out. There was no problem of multicollinearity in the analyses, as supported by a low variance inflation ratio (<1.32).

**Table 4:** **The findings of regression analyses for participants' performance in the Simon task after partialling out the participants' age (i.e., cognitive maturation)**.

		***R^2^* change^a^**	**L2 proficiency**			***R^2^* change^b^**	**L2:L1 ratio**		
			***beta***	***t*_(93)_**	***p***		***beta***	***t*_(93)_**	***p***
Overall	RT	0.01	−0.13	1.38	0.09	0.02	−0.15^*^	1.80	0.04
	Error	<0.001	0.01	0.10	0.46	0.01	0.09	0.88	0.19
	μ	0.01	−0.09	0.96	0.17	0.01	−0.13	1.43	0.08
	σ	0.01	−0.10	0.88	0.19	0.02	−0.13	1.29	0.10
	τ	0.01	−0.12	1.15	0.13	0.01	−0.12	1.31	0.10
Simon Effect	RT	0.02	−0.14	1.24	0.11	0.03	−0.20^*^	1.86	0.04
	Error	0.001	−0.04	0.31	0.38	0.004	0.07	0.62	0.27
	μ	0.04^*^	−0.22^*^	1.88	0.03	0.05^*^	−0.25^*^	2.35	0.01
	σ	0.02	−0.14	1.25	0.11	0.02	−0.15	1.41	0.08
	τ	0.01	0.12	1.01	0.16	0.01	0.10	0.87	0.20
Congruent Trials	RT	0.01	−0.11	1.23	0.11	0.02	−0.13	1.56	0.06
	Error	0.001	0.04	0.36	0.36	0.003	0.05	0.52	0.31
	μ	0.003	−0.06	0.63	0.27	0.01	−0.11	1.15	0.13
	σ	0.002	−0.05	0.45	0.33	0.01	−0.09	0.91	0.19
	τ	0.01	−0.13	1.32	0.10	0.01	−0.11	1.21	0.12
Incongruent Trials	RT	0.02^*^	−0.16^*^	1.72	0.045	0.03^*^	−0.20^*^	2.32	0.01
	Error	<0.001	−0.01	0.08	0.47	0.01	0.09	0.88	0.19
	μ	0.03^*^	−0.21^*^	2.19	0.02	0.06^*^	−0.27^*^	3.12	0.00
	σ	0.03^*^	−0.20^*^	1.77	0.04	0.05^*^	−0.25^*^	2.36	0.01
	τ	0.001	−0.03	0.31	0.38	0.001	−0.03	0.32	0.38

* *p < 0.05 (one-tailed). Please refer to Table 2 for the means and standard deviations of different measures. The R^2^ change^a^ column shows the increase in R^2^ after adding L2 proficiency predictor variables alone and its significance. The R^2^ change^b^ column shows the increase in R^2^ after adding L2:L1 ratio predictor variables alone and its significance. The L2 proficiency and L2:L1 ratio columns show the significance of the L2 proficiency and L2:L1 ratio predictor variables in the regression analyses, respectively*.

**Table 5:** **Mean statistics and findings of regression analyses for participants' performance in the Simon switching task after partialling out the participants' age (i.e., cognitive maturation)**.

		***R^2^* change^a^**	**L2 Proficiency**			***R^2^* change^b^**	**L2:L1 ratio**		
			***beta***	***t*_(93)_**	***p***		***beta***	***t*_(93)_**	***p***
Overall	RT	0.001	−0.04	0.37	0.36	0.02	−0.13	1.40	0.09
	Error	0.01	−0.08	0.74	0.23	0.01	0.11	0.98	0.17
	μ	0.003	−0.06	0.55	0.29	0.03^*^	−0.18^*^	1.88	0.03
	σ	0.01	−0.09	0.78	0.22	0.02	−0.13	1.24	0.11
	τ	<0.001	<0.001	0.001	0.50	0.001	−0.03	0.32	0.38
All trials in the pure block	RT	0.002	−0.06	0.55	0.29	0.02	−0.14	1.50	0.07
	Error	0.01	−0.10	0.91	0.19	0.01	0.07	0.69	0.25
	μ	0.001	−0.04	0.40	0.35	0.02^*^	−0.16^*^	1.71	0.45
	σ	0.002	−0.05	0.45	0.33	0.01	−0.09	0.85	0.20
	τ	0.002	−0.05	0.47	0.32	0.003	−0.06	0.63	0.27
All trials in the mixed block	RT	<0.001	0.01	0.06	0.48	0.01	−0.10	0.98	0.17
	Error	0.002	−0.05	0.44	0.33	0.01	0.10	0.95	0.18
	μ	0.001	0.03	0.23	0.41	0.02	−0.15	1.41	0.08
	σ	<0.001	0.01	0.10	0.46	0.10	−0.10	0.95	0.18
	τ	<0.001	−0.02	0.17	0.44	<0.001	0.02	0.19	0.43
Switch trials in the mixed block	RT	0.001	0.03	0.30	0.39	0.01	−0.08	0.84	0.21
	Error	<0.001	−0.02	0.17	0.44	0.02	0.13	1.29	0.10
Nonswitch trials in the mixed block	RT	<0.001	−0.01	0.09	0.47	0.01	−0.10	1.02	0.16
	Error	0.004	−0.07	0.64	0.27	0.003	0.06	0.53	0.30
Global switch cost	RT	0.002	0.05	0.44	0.33	<0.001	−0.01	0.08	0.47
	Error	0.001	0.03	0.29	0.39	<0.001	−0.02	0.17	0.44
Local switch cost	RT	0.01	0.10	0.84	0.20	<0.001	0.02	0.19	0.43
	Error	0.004	0.08	0.67	0.26	0.01	0.11	1.03	0.15

* *p < 0.05 (one-tailed). Please refer to Table 3 for the means and standard deviations of different measures. The R^2^ change^a^ column shows the increase in R^2^ after adding L2 proficiency predictor variables alone and its significance. The R^2^ change^b^ column shows the increase in R^2^ after adding L2:L1 ratio predictor variables alone and its significance. The L2 proficiency and L2:L1 ratio columns show the significance of the L2 proficiency and L2:L1 ratio predictor variables in the regression analyses, respectively*.

#### Simon task

**Figure 1:**
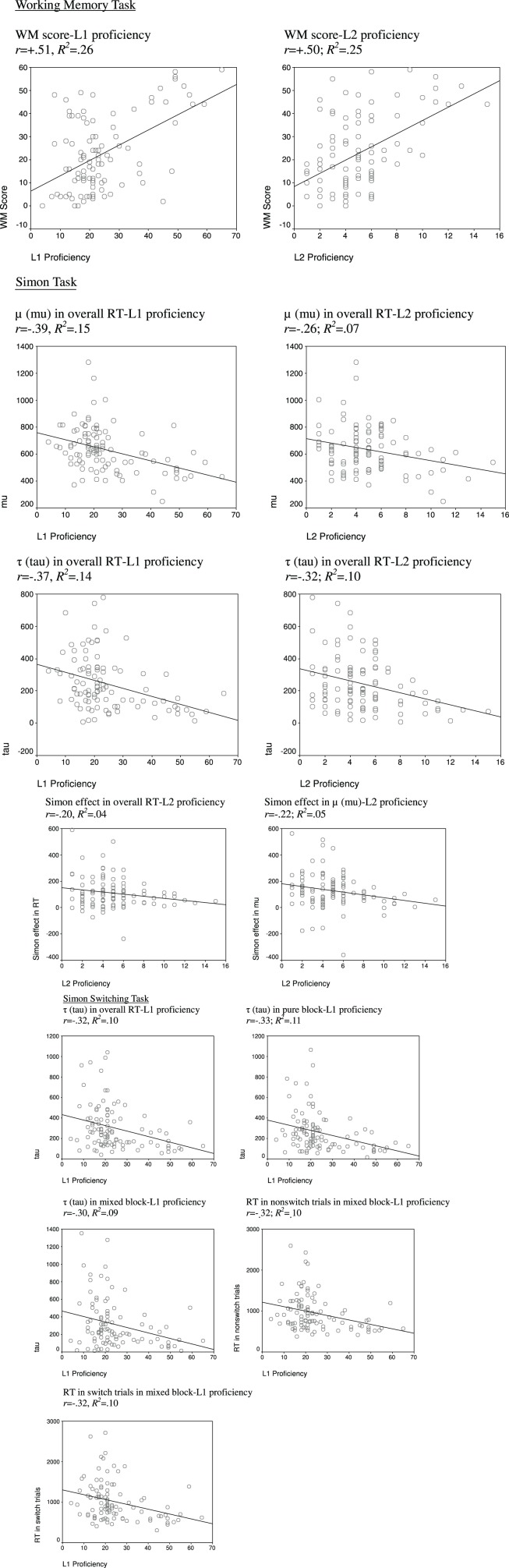
**Scatterplots for the relationship between dependent measures and bilinguals' L1 or L2 proficiency**. (*Note*: *r* and *R^2^* are based on Pearson correlation analyses and the model fit of linear regression, respectively. See the main text for a potential complication for the interpretation of the findings of L1 proficiency due to its strong correlation with participants' age).

As summarized in Table 2, bilinguals with higher L1/L2 proficiency showed faster RT than those with low L1/L2 proficiency. Analyses on ex-Gaussian parameters that were estimated by RT collapsed across congruent and incongruent trials revealed that the benefit of L1/L2 proficiency in overall RT was due to a reduction in both μ and τ. The Simon effect in RT could be predicted by L2 proficiency (see Figure 1), but not by L1 proficiency. Analyses on ex-Gaussian parameters showed that the benefit of L2 proficiency in the Simon effect was driven by the Simon effect in μ, but not the Simon effect in τ. We obtained similar findings when we fit the regression model for RT, μ, and τ in incongruent trials, after controlling for RT, μ, and τ in congruent trials and other extraneous variables. These analyses yielded significant effects of L2 proficiency for RT [*beta* = −0.09, *t*_(92)_ = 1.86, *p* = 0.035] and μ [*beta* = −0.23, *t*_(92)_ = 3.02, *p* < 0.01], but not for τ [*beta* = 0.05, *t*_(92)_ = 0.60, *p* = 0.28]. When participants' RT, μ, and τ in congruent and incongruent trials were separately fitted into the regression models, the results were all in the predicted direction and consistent with those reported for overall RT, even though not all of the effects approached significance (e.g., the relationship between L2 proficiency and μ in congruent trials, see Table 2).

After participants' age was partialled out, the Simon effect in RT and in μ, and RT and μ in incongruent trials were still significantly predicted by L2 proficiency (and L2:L1 ratio), while τ in overall RT was no longer predicted by any L2 proficiency indicator (see Table 4). When we fit the regression model for RT, μ, and τ in incongruent trials, after controlling for RT, μ, and τ in congruent trials and other extraneous variables (including participants' age), the results were similar though not all effects remained significant. The analyses yielded significant effects of L2 proficiency for μ [*beta* = −0.17, *t*_(92)_ = 2.27, *p* = 0.01], but not for RT [*beta* = −0.06, *t*_(92)_ = 1.30, *p* = 0.10] or τ [*beta* = 0.06, *t*_(92)_ = 0.75, *p* = 0.24], and significant effects of L2:L1 ratio for RT [*beta* = −0.08, *t*_(92)_ = 1.94, *p* = 0.03] and μ [*beta* = −0.21, *t*_(92)_ = 3.01, *p* < 0.01], but not for τ [*beta* = 0.05, *t*_(92)_ = 0.64, *p* = 0.53].

Overall, the results were apparently in line with Tse and Altarriba (2012) that the effect of L2 proficiency occurred in both conflict resolution (as reflected by Simon effect in μ) and goal maintenance (as reflected by τ). However, after taking into account the cognitive maturation by partialling out the effect of participants' age, the effect of L2 proficiency only occurred in conflict resolution, but not in goal maintenance. Moreover, L2 proficiency (and L2:L1 ratio) predicted only the RT/μ in incongruent trials but not the RT/μ in congruent trials, indicating that the effect of L2 proficiency was most salient when the task demanded conflict resolution. This suggests that the apparent effect of L2 proficiency on goal maintenance could be attributed to participants' cognitive maturation, rather than the effect of language proficiency *per se*.

#### Simon switching task

Unlike the Simon task, the findings of the Simon switching task were not as clear-cut. First, as summarized in Table 3, none of the dependent measures in this task was predicted by bilinguals' L2 proficiency. Second, bilinguals' L1 proficiency significantly predicted their overall RT, and RT in the pure and mixed blocks, which were driven by τ (see Figure 1), rather than μ. Third, bilinguals' L1 proficiency also significantly predicted their RT in the nonswitch and switch trials within the mixed block. Fourth, neither bilinguals' L1 nor L2 proficiency predicted their global or local switch cost in our children sample, which was not consistent with previous studies that involved a college-age population (e.g., Prior and MacWhinney, 2010; Hernández et al., 2013). After participants' age was partialled out, only the μ in overall RT and in the pure block was significantly predicted by L2:L1 ratio (see Table 5). Overall, these results suggested that the effect of L2 proficiency on switching performance was very weak and not systematic in bilingual children.

#### Working memory task

Both L1 and L2 proficiencies significantly predicted bilinguals' WM scores [*beta* = +0.35, *t*_(93)_ = 3.56, *p* < 0.01, and *beta* = +0.33, *t*_(93)_ = 3.57, *p* < 0.01, respectively]. Bilinguals with higher L1/L2 proficiencies showed higher WM scores in the operation span task (see Figure 1). After participants' age was partialled out, bilinguals' L2 proficiency and L2:L1 ratio still significantly predicted their WM scores [*beta* = +0.31, *t*_(93)_ = 3.52, *p* < 0.01, and *beta* = +0.16, *t*_(93)_ = 1.87, *p* = 0.04, respectively]. This suggests the relationship between L2 proficiency and working memory was not significantly compromised by the effect of cognitive maturation.

## Discussion

In the current study, we investigated the influences of bilingual children's L1 and L2 proficiencies on performance in three tasks that tap attentional control abilities: the Simon task, the Simon switching task, and the operation span task. Following some recent studies (e.g., Tse and Altarriba, 2012), we examined the RT data at the mean and distributional levels. We took into account participants' socioeconomic status and nonverbal intelligence to reduce their extraneous influence and used nonverbal Simon and Simon switching tasks to minimize linguistic influences. We repeated the same sets of analyses, with participants' age being controlled for, to investigate whether the relationship between L2 proficiency and attentional control remained significant after taking into account the potential effect of cognitive maturation.

Before summarizing the major findings of the three tasks, with elaborations on their implications, it is important to note a potential issue related to L1 proficiency. Given the strong correlation between L1 proficiency and participants' age (+0.80), the relationship between L1 proficiency and attentional control might likely be modulated by participants' cognitive maturation. That is, it is difficult to tease apart the influence of L1 proficiency and cognitive maturation on bilinguals' attentional control. Hence, the effects of L1 proficiency we obtained in the current study likely reflected the combined influence of native-language proficiency and cognitive maturation.

### Findings of the Simon task and their implications

First, bilingual children responded faster as their L1 and/or L2 proficiency increased (see Table 2), consistent with previous studies that defined full vs. partial bilinguals only by L2 proficiency (e.g., Carlson and Meltzoff, 2008), as well as the studies targeting bilingual vs. monolingual differences (see, e.g., Hilchey and Klein, 2011, for a review). After controlling for participants' age, the relationship between L2 proficiency and overall RT was significant in the incongruent trials, but not in the congruent trials. Because participants need to resolve the conflict between the arrow's direction and arrow's location in the incongruent trials, but not in the congruent trials, the *pure* effect of L2 proficiency occurred only when the task demanded more conflict resolution (see Table 4).

Second, the Simon effect in RT decreased as a function of bilinguals' L2 proficiency but not as a function of their L1 proficiency (see Table 2). After taking into account the effect of cognitive maturation (i.e., participants' age), this variable was significantly predicted by bilinguals' L2:L1 ratio (see Table 4). This was consistent with most of the previous studies that focused on the monolingual vs. bilingual comparison in children (e.g., Bialystok et al., 2008) as long as monolinguals can be regarded as bilinguals with *no* knowledge in L2 (i.e., the least L2-proficient bilinguals). However, the absence of a relationship with L1 proficiency in the current child population was contrary to Tse and Altarriba (2012), who reported a unique contribution of L1 proficiency on the reduction of the Stroop effect in a college student population. However, besides the difference in participant populations, the operational definition and homogeneity of L1 proficiency was different in the two studies: while it was homogeneous across participants measured by a standardized test in the current study, it was varied across participants and defined by self-reported ratings in Tse and Altarriba. Besides, the interpretation of the L1 proficiency findings may be complicated by its strong association with participants' age. Hence, it is possible that some, if not all, of these factors might contribute to the discrepancy between the two studies.

Third, by partitioning participants' overall RT and Simon effects (a RT slowdown in incongruent trials, relative to congruent trials) into ex-Gaussian parameters in the Simon task, we found that L2 proficiencies modulated τ in overall RT that reflects goal maintenance and the Simon effect in μ that reflects conflict resolution (see Table 2). Although bilinguals' L1 proficiency was associated with τ in overall RT, but not with the Simon effect in μ, this would be due to the effect of cognitive maturation. After controlling for participants' age, only the influence on the Simon effect in μ remained significant, suggesting that the apparent influence of L2 proficiency on goal maintenance was only due to the effect of cognitive maturation (see Table 4).

The findings that bilingual children's L2 proficiency was only associated with the Simon effect in μ, but not τ in overall RT, were not consistent with Tse and Altarriba (2012), who reported the effect of L2 proficiency in both conflict resolution and goal maintenance in the Stroop task. These results were also not in line with Calabria et al. (2011), who reported that the bilingual vs. monolingual difference on the congruency effect lay on τ, rather than in μ, in the flanker task. It is noteworthy that according to Kornblum's (1994) Dimensional Overlap Model (see also Blumenfeld and Marian, 2014), attentional control demanded in the arrow Simon task is similar to that in the Stroop and flanker tasks, so performance in these tasks should be quite comparable. Furthermore, the null influence of L2 proficiency on goal maintenance was not compatible with the findings of the enhanced monitoring abilities in bilinguals (vs. monolinguals) demonstrated in other tasks (e.g., Attention Network Task in Costa et al., 2009). Note that the equal proportion of congruent and incongruent trials (50:50) in the current arrow Simon task would be demanding enough for the participants to keep the task goal in mind across trials during the task. Nevertheless, all these studies involved college-age participants, so it is possible that the relationship between L2 proficiency and goal maintenance was salient only when the attentional control system has fully been developed (but see below for an alternative explanation).

In summary, we showed that relative to those with lower L2 proficiency, bilinguals with higher L2 proficiency possessed stronger abilities in conflict resolution in the Simon task. This is congruent with the idea that bilinguals need to select the suitable language schema (e.g., Green, 1998) before proceeding to subsequent lexical processing during a conversation. More explicitly, the way in which bilinguals resolve the lexical competition between languages is analogous to the demands of conflict resolution between the arrow's direction and the arrow's location in the Simon task. The more experience they have in resolving the lexical competition, the stronger conflict resolution abilities they may show in the Simon task. The absence of the relationship between L2 proficiency and goal maintenance could be attributed to the fact that bilingual children have not yet fully developed their attentional control system. Another possible explanation is that most of the participants in the current study were beginning L2 learners and the majority of child-parent conversation at home was in their L1, Cantonese, such that they did not have much experience deciding which language had to be prioritized during a conversation. As a result, the degree to which these participants were able to monitor the task goal was not as likely associated with their L2 proficiency, as with their cognitive maturation, which was indirectly quantified by their age.

### Findings of the Simon switching task and their implications

The findings in the Simon switching task were different from those reported in the literature. While we found expected negative relationships between language proficiency and the overall RT/τ in this task, they occurred only for L1 proficiency, but not for L2 proficiency (see Table 3). However, the faster RT/smaller τ could also be due to cognitive maturation as a function of participants' age. More importantly, while we did obtain significant global and local switch costs across participants, which demonstrated that the Simon switching task did yield typical findings reported in previous task switching studies, neither of these costs was associated with bilinguals' L1/L2 proficiencies. These results remained unchanged after taking into account participants' age. This was inconsistent with previous findings that bilinguals showed smaller local switch costs (e.g., Prior and MacWhinney, 2010) or smaller global switch costs (e.g., Hernández et al., 2013) than monolinguals. However, it is noteworthy that previous studies involved college-age bilinguals and few studies have examined the relationship between L1/L2 proficiency and switch costs in a bilingual child population. The current findings of local (or global) switch cost could suggest that the relationship between L1/L2 proficiencies and task-set switching abilities (or goal maintenance) was absent in a bilingual child population. This null finding could be explained as follows. Because most of our participants were beginning L2 learners and the majority of child-parent conversation at home was in their L1, Cantonese, they presumably did not have too much experience in switching codes between two languages during their daily-life conversation. Therefore, their task-set switching abilities were not sensitive to their L2 proficiencies. This *post-hoc* explanation should be validated by further experimentation. Besides, before accepting the null findings, two cautionary notes should be made for the interpretation of these results.

First, since the bilingual children recruited in the current study were high in mean nonverbal intelligence (see Table 1), one could question whether this might have masked the relationship between L2 proficiency and task switching performance. That is, all participants in our study might be so well-functioning that their task switching performance was already at ceiling level and thus not sensitive to the variation in their L2 proficiency. However, if this were the case, it would not be clear why L1/L2 proficiency could predict performance significantly in the simpler task (i.e., Simon task) in the same group of participants. Nevertheless, it would be better to test bilingual children with a wider range of nonverbal intelligence to examine whether the current findings could be generalizable across them.

Second, although the measures being used have all been widely used in the literature, we defined our bilingual children's L1/L2 proficiency mainly by their vocabulary knowledge in the current study. It could be argued that the global and local switch costs in the Simon switching task were not sensitive to children's vocabulary knowledge. Also, we did not ask participants (or their parents) about their frequency of usage for the two languages or the frequency of code switching between them (see Prior and Gollan, 2011; Soveri et al., 2011; for examples). It was possible that the extent to which their high L1/L2 vocabulary knowledge did not necessarily reflect how frequently they switch languages in daily life and therefore was not sensitive to participants' global or local switch costs. Future studies should include this critical measure and examine whether bilinguals' local and global switch costs in the Simon switching task were associated with how frequently they typically switch languages in daily life.

### Findings of the operation span task and their implications

Bilingual children's L1 and L2 proficiency were positively associated with WM scores, showing a relationship between bilinguals' language proficiencies and their working memory capacities. The relationship between L2 proficiency and WM scores remained significant after controlling for participants' age. This was consistent with previous studies that examined the superiority of bilinguals in resisting distractions from working memory load (e.g., Morales et al., 2013a). To our knowledge, the current study is the first to report positive relationships between bilinguals' L1/L2 proficiency and working memory capacity in a standard complex memory span task, which has widely been used in other research areas (e.g., Tse and Pu, 2012).

As mentioned in the Introduction Section, our operation span task has a relatively lower language requirement than the reading span task, making it more likely to reflect bilinguals' genuine working memory capacity (e.g., Sanchez et al., 2010). However, one could argue that our task may not provide a *pure* nonverbal working memory measure because it involved memorization of letter sequences and their repetition after arithmetic problems. While individual variation in the knowledge of English alphabets is likely much smaller than overall English (L2) proficiency, we have to acknowledge that part of the variances in the WM scores could be accounted for by participants' proficiency in English alphabets. We are now conducting a study to clarify the relationship between bilingualism and different types of working memory measures, including nonverbal tasks like symmetry span in Unsworth et al. (2005). In future studies, bilinguals' working memory capacities could also be assessed via some standardized measures (e.g., St. Clair-Thompson, 2014) to reveal a more comprehensive picture for the relationship between bilingualism and working memory.

Another contribution to the literature is related to our examination of the relationship between various measures in attentional control and working memory capacity. First, consistent with studies conducted with non-bilingual populations (e.g., Tse et al., 2010), the tail of the RT distribution in the Simon and Simon switching tasks (τ) were more associated with working memory capacity than the modal portion of the RT distribution (μ and σ). Second, although it seems to be reasonable to assume that those who have larger working memory capacities were better able to keep two task sets in mind and in turn showed less global switch cost, the current study actually showed a null correlation between global switch cost and working memory capacity. Rather than working memory capacities, global switch costs were correlated with τ in congruent trials in the Simon task and τ in the pure block of the Simon switching task. This could suggest that goal maintenance plays a larger role than working memory when participants need to hold two task sets in mind during the switching task. All these findings were obtained after controlling for participants' age. However, these results were found in bilingual children. Given that the relationships among attentional control measures might not be necessarily the same in bilinguals and monolinguals and across ages, future studies should further validate them by using monolingual samples with other age groups (e.g., college students).

Overall, based on the findings of the Simon, Simon switching, and operation span tasks performed by Cantonese-English bilingual children, we found that bilinguals' L2 proficiency modulated task performance by affecting conflict resolution and working memory. Although there was some evidence for the relationship between L1/L2 proficiency and goal maintenance, those findings were all compromised by the concurrent effect of cognitive maturation, as quantified by participants' age. The task-set switching abilities, as reflected by the local switch cost, were not modulated by bilinguals' L1 or L2 proficiencies, at least when they were defined by their vocabulary knowledge on standardized tests.

### Implications of the current findings on other theoretical accounts in the bilingual advantage

Apart from the constructs (conflict resolution, goal maintenance, task-set switching, and working memory) that we considered in the current study, the current findings could also shed light on other accounts of bilingual advantages, even though our study was not designed to test them. According to Miyake et al. (2000), attentional control constitutes three distinct, yet inter-related components: mental set shifting (or cognitive flexibility), updating (or working memory), and response inhibition. Previous findings that bilinguals could be more superior to monolinguals in some aspects of attentional control could be attributed to their greater experience in monitoring and updating language cues, inhibiting irrelevant and inappropriate language, and switching to the appropriate language in the communicative context. Given that the current four constructs could be mapped onto this account: mental set shifting ↔ task-set switching, updating ↔ goal maintenance and working memory, and response inhibition ↔ conflict resolution, our findings suggest that not all of the components in Miyake et al.'s attentional control framework were equally sensitive to bilinguals' L1 and L2 proficiencies; for example, bilinguals' L2 proficiency seems to modulate their response inhibition to a greater extent than mental set shifting.

Similar to Miyake et al.'s (2000) and our current conceptualizations, other researchers (e.g., Colzato et al., 2008; Costa et al., 2009; Bialystok et al., 2012) proposed that the bilingual advantage may better be explained by a dynamic combination of proactive (monitoring) and reactive (inhibition) control mechanisms, akin to Braver's (2012) dual-mechanism framework of cognitive control, rather than by a single mechanism. They argue that the two mechanisms are relatively independent and complementary in modulating attentional control. While proactive control facilitates attentional control by triggering the task goal in preparation and maintaining those processes during periods in which they are required, it demands working memory resources to keep the task goal continuously active while performing the task. This process could be conceptualized as the monitoring that bilinguals need to do prior to selecting the appropriate language for comprehension and production in particular contexts. In contrast, reactive control does not demand working memory resources, although the task goal has to be reactivated and conflict detection mechanisms are required to signal when suppression is needed. The current findings show that after controlling for the effect of cognitive maturation, only conflict resolution, which is analogous to the reactive control mechanism, but not goal maintenance, which is analogous to the proactive control mechanism, was modulated by bilinguals' L2 proficiency. Moreover, the presence of the correlation between working memory capacity and τ and the absence of the correlation between working memory capacity and the Simon effect in μ in the Simon task were in line with the view that the proactive control mechanism demands working memory resources, whereas the reactive control mechanism does not. In short, the current findings could be accommodated by other accounts, despite the fact that they have originally been proposed to explain the bilingual vs. monolingual difference in tasks that demand attentional control.

## Conclusion

Using three cognitive tasks that demand various types of attentional control abilities and RT analyses at both mean and distributional levels, the current results suggest that bilingual children's L2 proficiency was associated with their conflict resolution and working memory capacity, but not goal maintenance or task-set switching, when they performed these cognitive tasks that demand attentional control. More research should be done within bilingual populations by taking into account both of their L1 and L2 proficiencies to shed light on the underlying processes involved in coordinating between two language systems and enhancing bilinguals' attentional control systems.

### Conflict of interest statement

The authors declare that the research was conducted in the absence of any commercial or financial relationships that could be construed as a potential conflict of interest.

## Footnotes

^1^ We repeated the regression analyses by entering the L1 × L2 proficiency interaction in the third step, but this predictor was not significant in any dependent measure, which was consistent with Tse and Altarriba (2012). Hence, we did not include this predictor variable in the final analyses.
